# An Unusual Cause of Abdominal Pain: Mesenteric Lymphadenopathy Secondary to Sarcoidosis Without Pulmonary Involvement

**DOI:** 10.7759/cureus.85797

**Published:** 2025-06-11

**Authors:** Brandon Wiggins, Chris Cenzer, John M Sullivan, Kyle Knight, Fady Banno, Nathan Landesman

**Affiliations:** 1 Gastroenterology, Henry Ford Health System, Grand Blanc, USA; 2 Family Medicine, Henry Ford Health System, Grand Blanc, USA; 3 Internal Medicine, Henry Ford Health System, Grand Blanc, USA; 4 Gastroenterology, Corewell Health William Beaumont Hospital, Royal Oak, USA

**Keywords:** gastrointestinal sarcoidosis, lymphadenopathy, mesenteric lymphadenopathy, non-caseating granulomas, sarcoidosis

## Abstract

Sarcoidosis is a systemic disease that affects multiple organs in the body but rarely affects the gastrointestinal (GI) tract. The symptoms of GI sarcoidosis may be nonspecific or silent. Often, it is discovered on computed tomography (CT) or esophagogastroduodenoscopy (EGD), and a biopsy is needed for diagnosis. Initial management is typically with prednisone; however, here, we present a rare case of GI sarcoidosis with mesenteric lymphadenopathy in the absence of pulmonary involvement, diagnosed via biopsy and treated successfully with methotrexate.

## Introduction

Sarcoidosis is defined as a multisystem disorder involving the presence of non-caseating granulomas in various organs [1]. While sarcoidosis predominantly affects the lungs (90%), extrapulmonary involvement occurs in up to 30% of cases, most commonly involving the skin, eyes, lymphatic system, and rarely the gastrointestinal (GI) system [2]. One hallmark finding of sarcoidosis involves perihilar lymphadenopathy, which is seen in up to 90% of patients in imaging studies [3]. However, peripheral lymphadenopathy is seen in up to 40% of patients with sarcoidosis. Approximately 30% of patients with sarcoidosis will present with intra-abdominal or mesenteric lymphadenopathy [4]. In this article, we will examine the case of a patient who developed GI symptoms secondary to mesenteric lymphadenopathy caused by biopsy-proven sarcoidosis without pulmonary involvement.

## Case presentation

**Figure 1:**
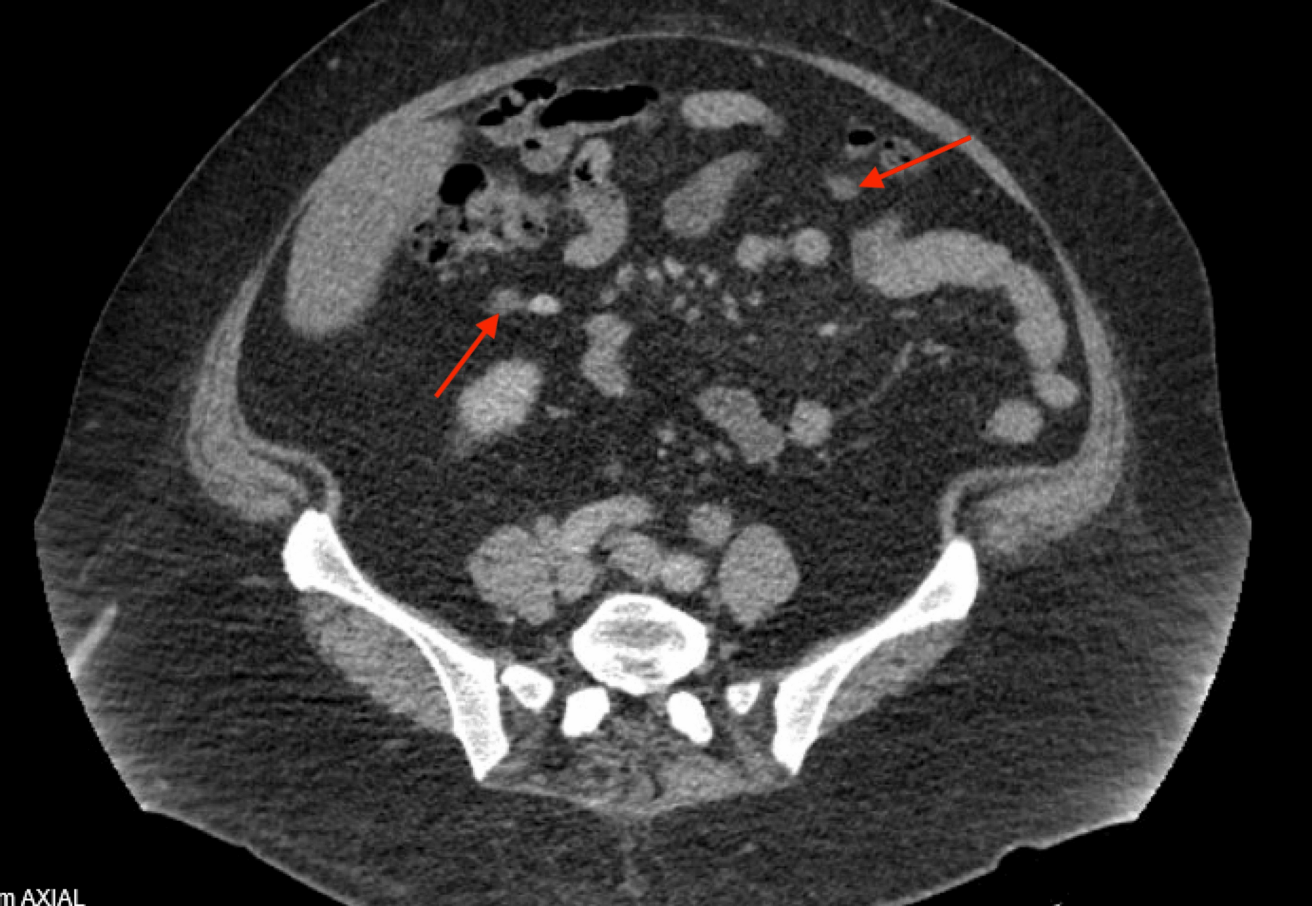
Initial CT scan of the patient's abdomen and pelvis showing evidence of mesenteric and retroperitoneal lymphadenopathy. CT, computed tomography

**Figure 2:**
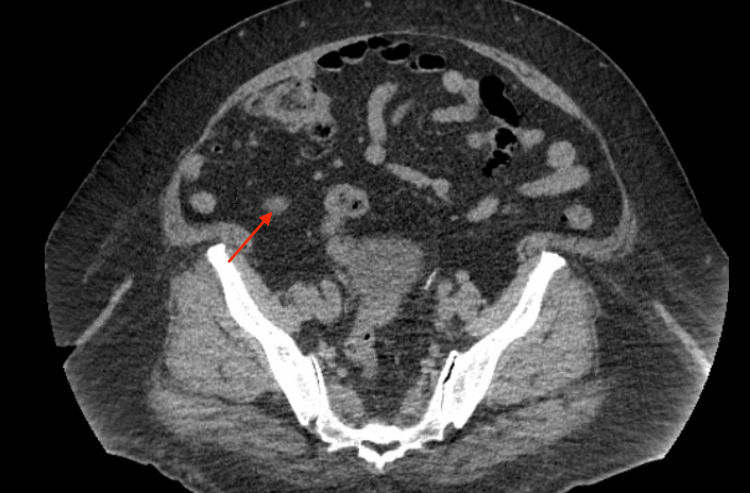
Second image of the patient’s initial CT scan of the abdomen and pelvis, showing evidence of mesenteric and retroperitoneal lymphadenopathy. CT, computed tomography

A 52-year-old female with a medical history of type 2 diabetes, pancreatic insufficiency, and obesity, and a surgical history of total hysterectomy with bilateral salpingo-oophorectomy and cholecystectomy, initially presented to the emergency room (ER) with complaints of recurrent abdominal pain. Of note, she was admitted to the hospital on multiple prior occasions for similarly presenting abdominal pain. She consistently described the pain as mostly in the right lower and upper quadrant, 10/10 on a pain scale, radiating to her right scapula. She reported that this pain had been gradually worsening over a two-week period and that she was no longer able to complete her daily activities of living (ADLs). She admitted to associated nausea and vomiting that were poorly controlled with oral medications as well as new-onset non-bloody diarrhea. Upon her initial presentation to the ER, she underwent a computed tomography (CT) scan of the abdomen and pelvis (Figure 1 and Figure 2), which revealed only mild mesenteric and retroperitoneal lymphadenopathy, likely representing an infectious, inflammatory, or neoplastic etiology. Her lab work was completely unremarkable without any evidence of transaminitis. Her presentation was ruled to be likely viral in nature, and she was sent home after her pain was controlled. 

**Figure 3:**
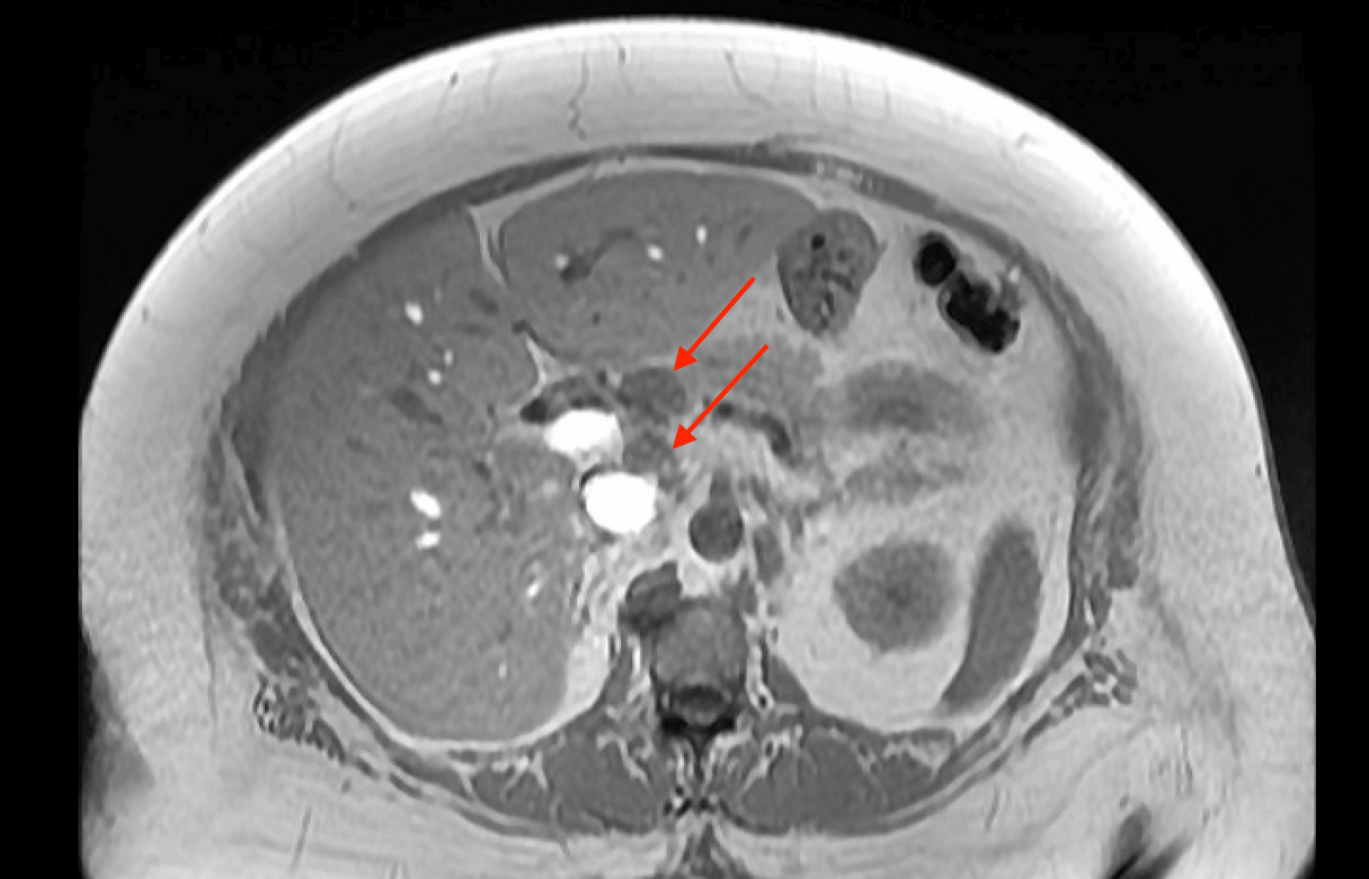
T1-weighted MRI showing peripancreatic lymphadenopathy.

**Figure 4:**
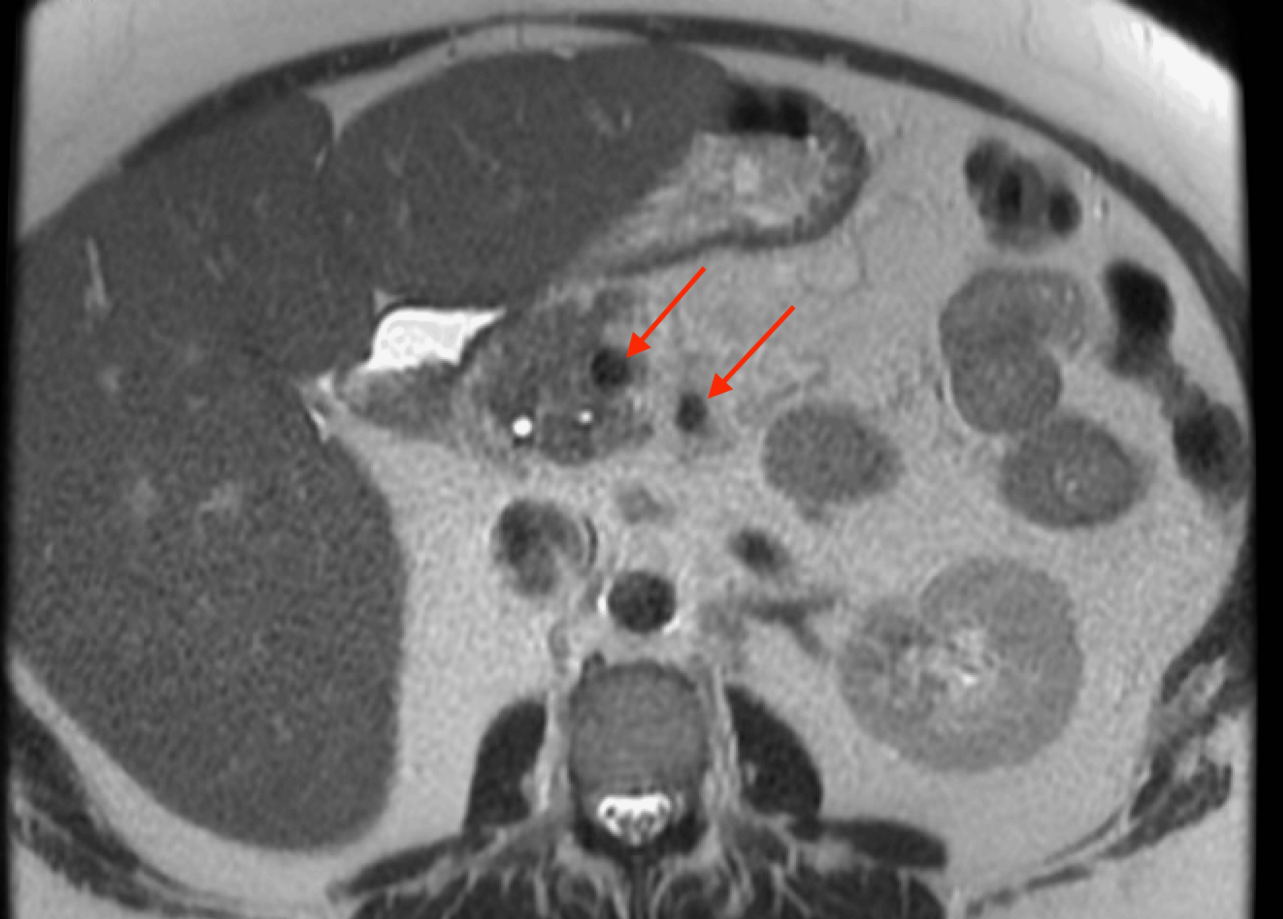
T2-weighted MRI showing peripancreatic lymphadenopathy.

**Figure 5:**
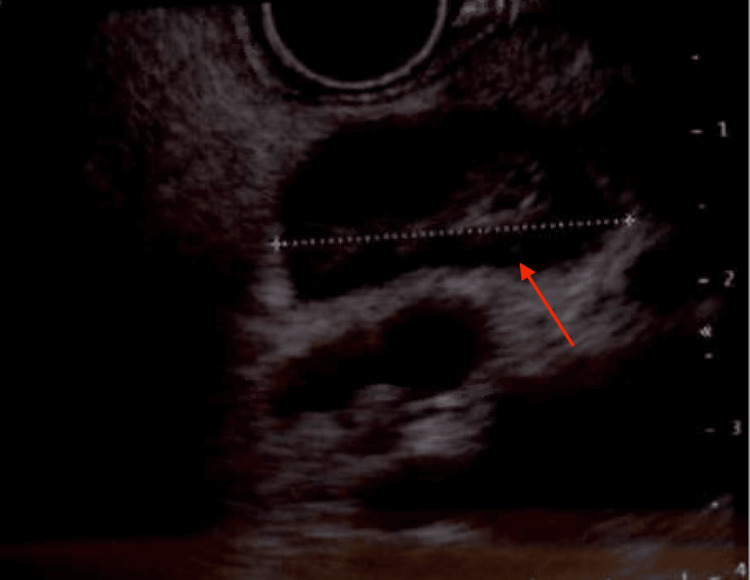
EUS showing an enlarged 2.3 cm perigastric lymph node. EUS, endoscopic ultrasound

Over the next few weeks, the patient started empiric treatment for suspected gastroparesis due to her comorbidities. Unfortunately, no success was seen in relieving her symptoms with Reglan and Zofran. On a subsequent hospital stay, she underwent an esophagogastroduodenoscopy (EGD). Findings showed no acute process as well as no signs of gastroparesis. It was recommended that she discontinue treatment as it had been unsuccessful. The patient’s pain during this time period was waxing and waning. Once her pain was controlled with low-dose Norco and non-steroidal anti-inflammatory medication, she was again discharged only to return to the hospital once again in severe pain. The patient required multiple modalities to determine and manage the etiology of her ongoing symptoms including but not limited to magnetic resonance cholangiopancreatography (MRCP, Figure 3 and Figure 4), EGD, dicyclomine, prednisone, and many different pain medications with no significant findings other than mesenteric lymphadenopathy or significant pain relief. Upon her gastroenterologist’s recommendation, she received an endoscopic ultrasound (EUS) with a biopsy (Figure 5) of her mesenteric lymph nodes, revealing noncaseating granulomas consistent with sarcoidosis.

After establishing a diagnosis of sarcoidosis based on her biopsy findings and clinical symptoms, her primary care physician initiated a trial of methotrexate as a potential treatment for the condition. After trialing this medication for multiple weeks with increasing doses, the patient reported decreased pain, nausea, and diarrhea. She admitted that the pain was not completely absent, but that it was no longer interfering with her ADLs. She was eventually referred to a sarcoidosis specialty clinic and continued to be followed on an outpatient basis. Her symptoms resolved with methotrexate, and the regimen was maintained by the specialty clinic.

## Discussion

The prevalence of sarcoidosis is between 50 and 160 per 100,000 people with an incidence of seven to 11 per 100,000 people in the United States of America [5,6]. Sarcoidosis occurs up to three times more in African Americans than Caucasian Americans and two times more likely in women [5,7]. 30% of cases of sarcoidosis have extrapulmonary manifestations with only 8% of these occurring without lung involvement [8,9]. Nearly 30% of patients with sarcoidosis have mesenteric lymphadenopathy, but the likelihood decreases to 10% when lymph nodes exceed 2 cm in size or when more than four nodes are involved [1].

Abdominal sarcoidosis including mesenteric lymphadenopathy can present with vague abdominal pain but is typically silent [10]. Most of abdominal sarcoidosis and lymphadenopathy is discovered on CT or EGD [1,11]. Sarcoidosis diagnosis requires a biopsy demonstrating non-caseating granulomas, typically performed on skin lesions or lymph nodes [11].

GI sarcoidosis including mesenteric lymphadenopathy that is symptomatic should be treated, especially if proven on biopsy with non-caseating granulomas [12]. Prednisone is the initial therapy of choice, with the recommended dose of 0.5 mg/kg each day, usually 30 to 40 mg. The dose can then be titrated down to 10-15 mg daily based on clinical improvement, with a reassessment of the need for continued treatment at the six-month mark [13]. An alternative agent that has been described in the literature, but usually only for hepatic sarcoidosis, is methotrexate. Methotrexate is used as a steroid-sparing agent in GI sarcoidosis, especially hepatic etiology with relatively similar success [14].

## Conclusions

GI sarcoidosis with concurrent mesenteric lymphadenopathy without pulmonary involvement is a rare manifestation of this systemic disease and presents with nonspecific symptoms, and diagnosis often requires a biopsy demonstrating non-caseating granulomas. Prednisone is the initial therapy of choice, but alternative agents such as methotrexate have also been shown to be effective. Prompt diagnosis and treatment are essential in managing symptoms and improving the quality of life of patients with this condition. Clinicians should maintain a high index of suspicion for sarcoidosis in patients presenting with vague abdominal pain and lymphadenopathy, especially in those with a history of the disease. Further research is needed to better understand the pathophysiology of GI sarcoidosis and to identify optimal treatment strategies.
